# The association between time scarcity, sociodemographic correlates and consumption of ultra-processed foods among parents in Norway: a cross-sectional study

**DOI:** 10.1186/s12889-017-4408-3

**Published:** 2017-05-15

**Authors:** Ingrid Laukeland Djupegot, Camilla Bengtson Nenseth, Elling Bere, Helga Birgit Torgeirsdotter Bjørnarå, Sissel Heidi Helland, Nina Cecilie Øverby, Monica Klungland Torstveit, Tonje Holte Stea

**Affiliations:** Department of Public Health, Sport and Nutrition, University of Agder, Postboks 422, 4604 Kristiansand, Norway

**Keywords:** Ultra-processed foods, Processed foods, Time scarcity, Convenience, Adults, Parents

## Abstract

**Background:**

Use of ultra-processed foods has expanded rapidly over the last decades and high consumption has been positively associated with risk of e.g. overweight, obesity and type 2 diabetes. Ultra-processed foods offer convenience as they require minimal time for preparation. It is therefore reasonable to assume that such foods are consumed more often among people who experience time scarcity. The main aim of this study was to investigate the association between time scarcity and consumption of ultra-processed foods among parents of 2-year olds in Norway. A secondary aim was to investigate the association between sociodemographic correlates, weight status and consumption of ultra-processed foods.

**Methods:**

This cross-sectional study included 497 participants. Chi-square and cross tabulations were used to calculate proportions of high vs. low consumption of ultra-processed foods in relation to time scarcity, sociodemographic correlates and weight status. Binary logistic regression analyses were performed to test the relationship between independent variables and consumption of ultra-processed foods.

**Results:**

Participants reporting medium and high time scarcity were more likely to have a high consumption of ultra-processed dinner products (OR = 3. 68, 95% CI = 2. 32–5.84 and OR = 3.10, 1.80–5.35, respectively) and fast foods (OR = 2.60, 1.62–4.18 and OR = 1.90, 1.08–3.32, respectively) compared to those with low time scarcity. Further, participants with medium time scarcity were more likely to have a high consumption of snacks and soft drinks compared to participants with low time scarcity (OR = 1.63, 1.06–2.49). Finally, gender, ethnicity, educational level, number of children in the household and weight status were identified as important factors associated with the consumption of certain types of ultra-processed foods.

**Conclusions:**

Results from the present study showed that time scarcity, various sociodemographic factors and weight status was associated with consumption of processed foods. Future studies with a longitudinal design are needed to further explore these patterns over a longer period of time.

**Electronic supplementary material:**

The online version of this article (doi:10.1186/s12889-017-4408-3) contains supplementary material, which is available to authorized users.

## Background

Highly processed foods have been classified as *ultra-processed* by Monteiro et al. in the NOVA^1^ food classification system, and include products that are industrially manufactured and usually highly accessible, attractive, palatable and habit-forming [1]. The NOVA classification categorizes foodstuffs in four groups based on the extent and purpose of processing; Unprocessed or minimally processed foods, processed culinary ingredients, processed foods, and ultra-processed foods (UPF) [1, 2]. Group 4 products (ultra-processed foods) are composed of industrial ingredients (e.g. corn syrup, lactose, soy proteins) and culinary ingredients that are refined or extracted from whole foods (e.g. flour, oil, sugar) [1, 2]. UPF are often referred to as convenience foods or fast foods, and examples include ready-meals, soft drinks, chocolate and chips [1]. Furthermore, UPF are typically energy-dense, low in dietary fibre, protein and micronutrients, and they often contain more sugar, sodium and fat/saturated fat than unprocessed and minimally processed foods [3–5]. An excess intake of UPF might therefore have severe implications for human health, and has been linked to several lifestyle related diseases including obesity, type 2 diabetes, metabolic syndrome, cardiovascular disease and cancer in children, adolescents and adults [6–16]. During the twentieth century, whole and fresh foods have increasingly been replaced with convenient pre-prepared and ready-to-eat products that require minimal preparation [3, 17]. A large prospective study conducted in ten European countries found that highly processed foods contributed with 61–79% of mean energy intake [18]. These results are consistent with findings from Canada and the United States, where approximately 60% of household food expenditure and mean energy intake was explained by purchasing and consumption of highly processed foods and beverages [19–21].

A range of factors might influence the use of UPF-products, and among these are time scarcity, which has previously been described as people’s perceptions or feelings of not having enough time to do all they want or need to in a day (Godbey, Lifset & Robinson, 1998, in Jabs & Devine 2006 p. 197) [22]. Families with children often operate on a tight schedule juggling work, domestic work and leisure activities [22, 23]. Qualitative studies have reported that employed mothers often experience a general lack of time, which also influence their food choices [23, 24]. Preparation of healthy foods was perceived to be a time-consuming activity, and thereby highly processed convenience foods were often used as a time saving strategy [23, 24]. Although qualitative research has indicated that feelings of time scarcity might contribute to less home-prepared meals with fresh ingredients and an increased use of e.g. ready-meals and fast foods, there is a lack of consistent quantitative evidence regarding this association [25]. As parents’ behaviour might influence the eating habits of their children [26, 27], investigating the influence of time scarcity on the use of convenience foods and fast foods among parents is of particular interest. Furthermore, quantitative studies have reported sociodemographic differences in ultra-processed food consumption (UPFC) [28, 29]. For illustration, low socioeconomic status has been associated with less healthy diets, including higher consumption of fast foods and soft drinks [30–33]. On this basis, it is appropriate to adjust for sociodemographic variables when investigating factors potentially influencing UPFC.

The main aim of this study was to investigate the association between time scarcity and UPFC among parents of 2-year olds in Norway. A secondary aim was to investigate the association between sociodemographic correlates (gender, ethnicity, education and number of children in the household), weight status and UPFC.

## Methods

### Design and study sample

This cross-sectional study is part of The Healthy and Sustainable Lifestyle project and the Child Food Courage project. Registration code for the Child Food Courage Project is 37,459. Data were collected between October 2014 and January 2015. The study was conducted in accordance with the Helsinki Declaration, and permission to collect and store data was obtained from Norwegian Social Science Data Services. Furthermore, written consent was electronically provided by the participants prior to data collection. About 3100 parents in the counties of Aust-Agder and Vest-Agder in Southern Norway, with children born in 2012, received information about the project through their kindergarten. Participants completed a web-based questionnaire (Additional file 1), which comprised a food frequency questionnaire and included questions about lifestyle behaviours, self-perceived health and quality of life among parents of toddler’s. In total, 605 parents signed up to participate. Only participants who completed the questionnaire (*n* = 497) were included in the current study, which yielded a response rate on approximately 16%.

### Outcome measures

Questions from the Healthy and Sustainable Lifestyle-survey were used to develop three scores to measure UPFC; Ultra-processed dinner products, sweet/salty snacks & soft drinks and fast foods away from home. The selection of questions was based on the NOVA classification of food products proposed by Monteiro et al. [1, 34]. There are currently no dietary recommendations regarding UPF in Norway that can inform the operationalisation of UPFC. For all outcome scores, cut-offs were therefore estimated to get the most equally sized groups to increase the statistical power, and variables were dichotomized into low vs. high consumption.

### Ultra-processed dinner products

This score comprised 5 items measuring frequency of consumption of ready-to-eat/pre-prepared dinner products. Questions included *How often do you eat … Noodles; Ready meals; Sausages; Pommes frites; Dinners based on minced meat (*e.g. *tacos, pasta).* Response alternatives were assigned different values, and ranged from *never* to *every day* (never = 0, less than once a month = 0.25, 1–3 times/month = 0.5, once a week = 1, 2 times/week = 2, 3 times/week = 3, 4 times/week = 4, 5 times/week = 5, 6 times/week = 6, every day = 7). Total score ranged from 0 to 35, with higher score indicating a higher consumption of ultra-processed dinner products.

### Sweet/salty snacks & soft drinks

This score comprised 4 items measuring frequency of consumption of sweet/salty snacks and soft drinks. Questions included *How often do you eat … Salted snacks (*e.g. *chips, cheese doodles, salted nuts); Confectionery (*e.g. *sweets, chocolate)*, and *How often do you drink … Sugar sweetened beverages (*e.g. *soft drinks, juice, ice tea, ice coffee); Artificially sweetened beverages (*e.g. *diet soft drinks, diet juice, diet ice tea)*. Response alternatives were assigned different values, and ranged from *never* to *several times a day* (never = 0, less than once a week = 0.5, once a week = 1, 2 times/week = 2, 3 times/week = 3, 4 times/week = 4, 5 times/week = 5, 6 times/week = 6, every day = 7, several times a day = 10). Total score ranged from 0 to 40, with higher score indicating a higher consumption of snacks & soft drinks.

### Fast foods away from home

This score comprised 2 items measuring frequency of consumption of food from fast food restaurants, gas stations and convenience stores. Questions included *How often do you eat food from fast food restaurants (*e.g. *McDonalds, takeaway-restaurants*) and *How often do you eat food bought at a gas station/convenience store (*e.g. *7-eleven)*. Response alternatives were assigned different values, and ranged from *never* to *every day* (never = 0, less than once a week = 0.5, once a week = 1, 2 times/week = 2, 3 times/week = 3, 4 times/week = 4, 5 times/week = 5, 6 times/week = 6, every day = 7). Total score ranged from 0 to 14, with higher score indicating a more frequent consumption of fast foods.

### Independent variables

#### Time scarcity

Van der Lippe’s adjusted version of Garhammer’s index of time pressure, was used to measure time scarcity [35, 36]. In this 7 item scale, study participants were asked to what extent the following statements coincided with their experiences: *I am under time pressure*, *I wish I had more time for myself*, *I feel I am under time pressure from others*, *I cannot deal with important things properly due to lack of time*, *I cannot get proper sleep*, *I cannot recover properly from illness due to lack of time* and *I am under so much time pressure that my health suffers*. Response alternatives ranged from *never* to *always* (1 = never, 2 = rarely, 3 = sometimes, 4 = often, 5 = always). Total score ranged from 7 to 35, with higher score indicating a higher degree of time scarcity. In previous studies, the time pressure scale has shown a high level of internal consistency, with a Cronbach’s alpha of 0.79 [35] and 0.87 [37]. Cut-offs were estimated to get the most equally sized groups, and the time scarcity variable was further trichotomized into a low, medium and high group.

#### Sociodemographic correlates and weight status

Gender (men vs. women), ethnicity (born in Norway vs. not born in Norway), educational level (higher education at university/college vs. no higher education), number of children in the household (2 vs. 1 and ≥3 vs. 1) and weight status (BMI ≥25.0 kg/m^2^ vs. ≤24.9 kg/m^2^) were also tested as possible predictors of UPFC. Participants with BMI ≥25.0 kg/m^2^ are referred to as overweight/obese, while participants with BMI ≤24.9 kg/m^2^ are referred to as normal weight as there were few underweight participants (BMI <18.5 kg/m^2^).

#### Statistical analyses

All analyses were performed with the statistical software package IBM SPSS Statistics version 22.0 (IBM Corp., Somers, NY, USA.). Proportions of study participants having a high consumption of ultra-processed dinner products, sweet/salty snacks & soft drinks and fast foods away from home, in relation to the proposed correlates, were calculated using cross tabulations and chi-square. Proportions classified with high time scarcity in relation to gender, ethnicity, educational level, number of children in the household and weight status were also calculated with cross tabulation and chi-square.

Binary logistic regression analyses were performed with appropriate sampling weights (for level of education and gender) to test the relationship between the independent variables (time scarcity, gender, ethnicity, educational level, number of children and weight status) and the dependent variables (consumption of ultra-processed dinner products, sweet/salty snacks & soft drinks and fast foods away from home). The variables were entered into the model in two blocks: First, the unadjusted relationship of time scarcity and UPFC was tested, and then the sociodemographic correlates and weight status were included in the model. Results were regarded significant at *p* < 0.05.

## Results

### Characteristics of study sample

In our final sample, 90% of study participants were women and 90% were born in Norway. Age ranged from 20 to 46 years (mean = 32.2 years), and mean Body Mass Index (BMI) was 24.9 kg/m^2^. Regarding educational status, 69% had higher education at university/college level (<4 years or ≥4 years). A total of 33% of the study participants had one child living in the household, 47% had two children, and 20% had three or more children.

### Time scarcity

In the present study, the time scarcity scale had a Cronbach’s alpha of 0.87. Mean score of experienced time scarcity in the study sample was 20.3 ± 5.1 (not reported in table). According to the results presented in Table 1, there were no differences between groups in the number of participants being categorized as having high degree of time scarcity.

**Table 1:** Descriptive statistics of the association between time scarcity, gender, age, ethnicity, weight status, education, number of children and dichotomized indicators of ultra-processed food consumption Percentage of groups in high category of time scarcity, ultra-processed dinner products, sweet/salty snacks & soft drinks and fast foods away from home. *p < 0.05

		Time scarcity	Ultra-processed dinner products	Snacks & Soft drinks	Fast foods away from home
	n	% in high category	% in high category	% in high category	% in high category
All	497	29.6	48.9	48.3	43.7
Time scarcity					
Low	157		43.3	43.9	35.7
Medium	193		49.7	49.2	49.2
High	147		53.7	51.7	44.9
p			0.183	0.380	0.037*
Gender					
Men	52	21.2	57.7	44.2	55.8
Women	445	30.6	47.9	48.8	42.2
p		0.160	0.180	0.536	0.063
Ethnicity					
Native	442	30.3	50.5	50.9	45.2
Non-native	54	24.1	37.0	25.9	29.6
p		0.343	0.063	0.001*	0.029*
Weight status					
Normal weight	293	29.0	46.8	45.1	40.6
Overweight/obese	197	29.9	52.8	54.3	49.2
p		0.823	0.190	0.044*	0.059
Education					
No higher education	153	28.1	53.6	56.9	51.0
Higher education	344	30.2	46.8	44.5	40.4
p		0.631	0.162	0.011*	0.028*
Number of children in the household					
1	163	28.2	47.2	48.5	44.8
2	231	30.7	48.1	47.2	42.9
≥3	101	28.7	53.5	51.5	44.6
p		0.849	0.580	0.771	0.918

(men vs. women, ethnic Norwegian vs. non-ethnic Norwegian, overweight/obese vs. normal weight, higher education vs. no higher education, 2/≥3 children vs. 1 child). In the descriptive analyses, 35.7% of participants with low time scarcity, 49.2% of participants with medium time scarcity and 44.9% of participants with high time scarcity were categorized as high consumers of fast foods (*p* = 0.037, Table 1). Weighted regression analyses adjusted for sociodemographic correlates and weight status showed that participants with both medium and high time scarcity were more likely to be high consumers of ultra-processed dinner products (OR = 3. 68, 95% CI = 2. 32–5.84 and OR = 3.10, 1.80–5.35, respectively) and fast foods (OR = 2.60, 1.62–4.18 and OR = 1.90, 1.08–3.32, respectively) compared to those with low time scarcity (Table 2). Further, participants with medium time scarcity, but not high time scarcity, were more likely to be high consumers of snacks & soft drinks compared to participants with low time scarcity (OR = 1.63, 1.06–2.49, Table 2).

**Table 2:** Odds ratios for the associations between time scarcity, sociodemographic correlates, weight status and high consumption of ultra-processed foods

	Ultra-processed dinner products				Snacks & Soft drinks				Fast food away from home			
	Model 1		Model 2		Model 3		Model 4		Model 5		Model 6	
	OR	95% Cl	OR	95% Cl	OR	95% Cl	OR	95% Cl	OR	95% Cl	OR	95% Cl
Time scarcity												
Medium (vs. low)	3.04*	2.01–4.59	3.68*	2.32–5.84	1.46	0.98–2.17	1.63*	1.06–2.49	2.19*	1.46–3.28	2.60*	1.62–4.18
High (vs. low)	2.66*	1.66–4.27	3.10*	1.80–5.35	1.36	0.86–2.16	1.57	0.94–2.61	1.62*	1.02–2.56	1.90*	1.08–3.32
Sex												
Men (vs. women)			2.58*	1.65–4.06			1.08	0.71–1.62			3.78*	2.35–6.08
Ethnicity												
Native (vs. non-native)			1.98*	1.11–3.53			1.87*	1.08–3.23			4.79*	2.50–9.15
BMI												
Overweight/obese (vs. normal weight)			1.54*	1.04–2.30			1.18	0.81–1.71			3.40*	2.26–5.11
Education												
Higher education (vs. no higher education)			0.61*	0.40–0.92			0.55*	0.37–0.81			0.68	0.45–1.04
Number of children in the household												
2 (vs. 1)			1.33	0.86–2.07			0.75	0.50–1.14			0.86	0.54–1.38
≥3 (vs. 1)			4.22*	2.26–7.89			1.75	1.00–3.08			1.53	0.82–2.85
Age												
Continous (years)			0.93*	0.89–0.97			0.98	0.94–1.02			0.90*	0.86–0.94

OR: Odds ratio; Cl: Confidence interval; *p < 0.05

### Sociodemographic correlates and weight status

Table 1 showed no difference in the number of male and female participants being categorized as having high consumption of ultra-processed dinner products, snacks & soft drinks and fast foods. Adjusted regression analyses, however revealed that men were more likely to be categorized as high consumers of ultra-processed dinner products and fast foods than women (OR = 2.58, 1.65–4.06 and OR = 3.78, 2.35–6.08, respectively).

A higher number of ethnic compared to non-ethnic Norwegians were categorized as having high consumption of snacks & soft drinks (50.9% vs 25.9%, *p* = 0.001) and fast foods (45.2% vs 29.6, *p* = 0.029) (Table 1), and the adjusted analyses confirmed that ethnic Norwegians were more likely to be categorized as high consumers of both ultra-processed dinner products (OR = 1.98, 1.11–3.53), snacks & soft drinks (OR = 1.87, 1.08–3.23) as well as fast foods (OR = 4.79, 2.50–9.15) compared to non-ethnic Norwegians.

Further, the results revealed that less participants with high education was categorized as having a high consumption of snacks & soft drinks (44.5% vs. 56.9%, *p* = 0.011) and fast foods (40.4% vs. 51.0%, *p* = 0.028) compared to those with low education (Table 1). According to adjusted regression analyses, high educated participants were less likely to be categorized as high consumers of both ultra-processed dinner products (OR = 0.61, 0.40–0.92) and snacks & soft drinks (OR = 0.55, 0.37–0.81) compared to those with low educational level (Table 2).

A higher number of overweight/obese participants was categorized as high consumers of snacks & soft drinks compared to normal weight participants (54.3% vs. 45.1%, *p* = 0.044, Table 1). Adjusted regression analyses did, however, not confirm an association between weight status and consumption of snacks & soft drinks. On the other hand, the adjusted analysed showed that overweight/obese participants were more likely to be categorized as high consumers of ultra-processed dinner products (OR = 1.54, 1.04–2.30) and fast foods (OR = 3.40, 2.26–5.11) than normal weight participants.

Furthermore, descriptive analyses did not show a significant difference between the number of participants with one, two or three children in the household being categorized as having a high consumption of ultra-processed dinner products, snacks & soft drinks and fast foods. Results from adjusted analyses showed, however, a higher consumption of ultra-processed food products among participants with 3 or more children in the household compared to those with one child in the household (OR = 4.22, 2.26–7.89).

## Discussion

### Time scarcity

Findings in the present study showed that time scarcity was associated with both consumption of ultra-processed dinner products, snacks & soft drinks as well as fast foods away from home after adjustment for sociodemographic correlates and weight status. Previously published studies have also suggested that time shortage is a barrier to healthy eating as those experiencing time pressure were more likely to eat fast food more frequently and less likely to meet the fruit and vegetable recommendations [38, 39]. In addition, time pressure, having a paid job and number of working hours have also been positively associated with the use of ready-meals such as frozen pizzas and TV dinners [40, 41], suggesting that use of ready-meals might be a convenient way of managing time pressure. The positive association between time scarcity and use of ultra-processed dinner products shown in the present study supports the hypothesis that the consumption of ready meals is used as a strategy for better time management.

### Sociodemographic correlates and weight status

In the current study, adjusted analyses showed that men were more likely to be classified as high consumers of ultra-processed dinner products and fast foods, but not snacks & soft drinks, when compared to women. These results have to some extent been supported by previous studies showing an increased consumption of fast foods, sugar sweetened soft drinks and processed meat and a decreased consumption of sweets among men compared to women, [29, 33, 42].

Furthermore, adjusted analyses in the present study showed that participants with high educational level had 39% lower odds of consuming ultra-processed dinner products and 45% lower odds of consuming snacks & soft drinks compared to those with low educational level. Whereas our study did not find a significant association between educational level and consumption of fast foods, Thornton et al. [31] found that low educational level, decreased household income and being a blue-collar employee was associated with an increase in fast food purchases. Similarly, Larson et al. [43] found that frequent fast food intake was most common among individuals with low-middle socioeconomic status, and another study has reported an association between dropping out of school before the age of sixteen and low levels of food involvement in the kitchen, resulting in a more frequent consumption of junk foods [44].

Results in the present study also showed higher odds of consuming both ultra-processed dinner products, snacks & soft drinks and fast foods among ethnic- compared to non-ethnic Norwegians. As processed foods tend to be less expensive than most fresh foods and ethnic minorities in Western societies often belong to low-income groups with lower living standards than the majority population [45], these findings were rather unexpected. Nevertheless, only 10% of the study sample was non-ethnic Norwegians and countries of origin for these participants were unknown. It is reasonable to assume that this small group was not representative of all non-ethnic parents in Norway, and furthermore there might be differences in UPFC in non-ethnic Norwegians with different countries of origin.

In the current study, a strong positive association was found between having three or more children in the household and consumption of ultra-processed food products, but not snacks & soft-drinks and fast foods, compared to having only one child in the household.

In contrast to our study, another study has confirmed that households with one child consumed more fast foods than households with no children and that households with more than one child consumed less fast foods than households with only one child [46].

Finally, adjusted analyses in the present study showed higher odds of consuming ultra-processed dinner products and fast foods, but not snacks & soft drinks, among overweight/obese participants compared to normal weight participants. A few previously published studies have assessed the potential association between weight status and intake of highly processed foods [6, 7, 12, 14], though the majority of these have investigated food intake as a predictor of overweight/obesity, rather than an outcome.

### Strengths and limitations

An important strength of the current study was the use of a validated measure on time scarcity. The use of three separate scores indicative of UPFC (ultra-processed dinner products, sweet/salty snacks & soft drinks and fast foods away from home) was also considered to be a strength, as it is reasonable to assume that there might be different factors influencing consumption of e.g. ready-meals and soft drinks. To the best of our knowledge, no previous studies have investigated the effect of time scarcity on UPFC, and the current study might therefore provide valuable input when developing future interventions and nutritional strategies. Nevertheless, there were also some study limitations. The response rate was only 16%, and the study sample was overrepresented by highly educated females. Due to the lack of representativeness in the study sample, analyses with appropriate sampling weights (for gender and educational level) were conducted.

Also, data collection was conducted in only two of Norway’s nineteen counties, thus findings in this study are not necessarily representative of Norwegian kindergarten parents in general. Regarding the operationalisation of UPFC, it is possible that other food classifications than NOVA could have been more appropriate. Also, the grouping of food items used as indicators of UPFC could have been more precise, especially for the score measuring consumption of fast foods away from home. Furthermore, the questionnaire was pilot-tested in seven subjects from a corresponding population of parents of toddlers, yet it is a limitation that the UPFC scores have not been validated. As we analysed cross-sectional data, conclusions regarding cause and effect cannot be drawn. Additionally, all data was based on self-report questionnaires, which might have increased the risk of social desirability bias. Considering sociodemographic, the variables were not tested independently, but entered as covariates in the logistic model.

## Conclusions

Findings in the present study suggest that time scarcity is associated with an increased consumption of UPF, including ultra-processed dinner products, snacks & soft drinks and fast foods, after adjusting for sociodemographic correlates and weight status. Furthermore, gender, ethnicity, educational level, number of children in the household and weight status were identified as important factors associated with the consumption of certain types of UPF. Future studies with a longitudinal design are warranted in order to further explore the relationship between time scarcity and UPFC.

## Additional file

Additional file 1: HSL Questionnaire. Questionnaire. English translation of the combined questionnaire from the cross-sectional studies The Healthy and Sustainable Lifestyle project and the Child Food Courage project. (ZIP 293 kb)

## Abbreviations

BMI: Body Mass Index
CI: Confidence interval
OR: Odds ratio
SD: Standard deviation
UPF: Ultra-processed foods
UPFC: Ultra-processed food consumption

## Footnotes

1 A name, not an acronym
